# Self-Induced Anaerobic Fermented Products of *Bacillus subtilis* M6 and *Lactiplantibacillus plantarum* R101 Improve Growth Performance in Broilers

**DOI:** 10.3390/ani16121754

**Published:** 2026-06-06

**Authors:** Yi-Tai Hsu, Kuo-Lung Chen, Ching-Chi Hung

**Affiliations:** 1 Ph.D. Program of Agriculture Science, National Chiayi University, Chiayi 600355, Taiwan; s1080004@mail.ncyu.edu.tw; 2 Department of Animal Science, National Chiayi University, Chiayi 600355, Taiwan; ckl@mail.ncyu.edu.tw; 3 Taiwan Livestock Research Institute, Ministry of Agriculture, Tainan 712009, Taiwan

**Keywords:** apparent total tract digestibility, animal nutrition, *Bacillus subtilis*, broiler, fermented product, growth performance, *Lactiplantibacillus plantarum*, nutrient digestibility, self-induced anaerobic fermentation, two-stage fermentation

## Abstract

This study compared a self-induced anaerobic fermented product (SIAFP), prepared with *Bacillus subtilis* M6 and *Lactiplantibacillus plantarum* R101, with dry-form (DTFP) and wet-form (WTFP) two-stage fermented products in broilers. Three trials were conducted: Trial 1 evaluated the physicochemical properties and growth performance; Trial 2 assessed nutrient composition and apparent total tract digestibility; and Trial 3 determined the optimal dietary inclusion level of SIAFP. In Trial 1, SIAFP exhibited the lowest pH and the highest *Lactiplantibacillus*-like counts (*p* < 0.05), and all fermented product groups showed higher body weight gain and performance efficiency factor compared to the unfermented control (*p* < 0.05). In Trial 2, SIAFP contained higher crude protein and total amino acid contents, concomitant with improved hemicellulose digestibility (*p* < 0.05). In Trial 3, incremental dietary inclusion of SIAFP (0–3.75%) exerted linear or quadratic effects on body weight gain and feed conversion ratio (*p* < 0.05), with optimal performance observed within the range of 1.25–2.5%. In conclusion, SIAFP showed comparable growth-promoting effects to DTFP and WTFP, suggesting its potential as a practical alternative fermented feed product. Dietary inclusion at 1.25–2.5% effectively enhanced growth performance, which may be attributed to improved nutrient composition and digestibility in broilers.

## Simple Summary

Two-stage fermented products have been shown to improve broiler growth performance; however, their production processes are time-consuming and require substantial equipment and energy inputs, thereby increasing production costs. This study evaluated a one-stage self-induced anaerobic fermented product (SIAFP) characterized by a shorter processing time and lower equipment requirements. Its physicochemical properties, nutrient composition, and effects on nutrient digestibility and growth performance in broilers were com-pared with those of dry-form (DTFP) and wet-form (WTFP) two-stage fermented products. The results indicated that the one-stage product exhibited greater acidification and higher nutrient digestibility. Its growth-promoting effects in broilers were comparable to those of two-stage fermented products, with an optimal dietary inclusion level of 1.25–2.5%. Overall, this technology effectively reduces production costs while maintaining growth performance, demonstrating strong potential as a practical and cost-effective fermented feed additive.

## 1. Introduction

The two-stage fermentation process, comprising an initial 2-day aerobic phase with *Bacillus subtilis*, followed by a 3-day anaerobic phase with lactic acid bacteria, has been shown to improve the growth performance of broiler chickens [1,2,3]. However, the drying process required for producing dry-form products is not only capital-intensive but may also compromise probiotic viability and the integrity of functional components [4,5,6]. Furthermore, the initial aerobic stage requires 2 days of processing and necessitates aeration, stirring, and environmental control equipment, thereby substantially increasing production costs. This stage is also frequently plagued by challenges such as elevated pH, off-odor generation, and excessive viscosity, which subsequently impede acidification.

Previous studies utilizing soybean meal and soybean hulls (7:3, *w*/*w*) as the fermentation substrate, both dry-form (DTFP) and wet-form (WTFP) two-stage fermented products, prepared with *B*. *subtilis* M6 in the first stage and *Lactiplantibacillus plantarum* R101 in the second stage, have been shown to enhance growth performance and economic benefits in broilers [7,8,9]. Nevertheless, the preparation of WTFP requires approximately 5 days, whereas DTFP requires 6–7 days, resulting in increased production costs. Therefore, shortening the fermentation time and reducing production costs are the primary objectives of this study.

Self-induced anaerobic fermentation technology has been widely applied in agricultural product processing and leverages the endogenous respiratory activity of raw materials or microbial metabolism to deplete oxygen, thereby establishing an anaerobic environment [10,11,12]. Compared with conventional fermentation processes, self-induced anaerobic fermentation eliminates the need for fermentation tanks or complex equipment, offering significant cost advantages. Moreover, it promotes the accumulation of organic acids and flavor compounds [13,14], thereby improving both product stability and economic viability. However, studies investigating the application of this technology in broiler diets remain limited.

It was hypothesized that SIAFP could improve nutrient utilization and growth performance in broilers compared with conventional two-stage fermented products, while reducing processing time and production costs. Therefore, its potential as a cost-effective alternative fermented product was evaluated in the present study.

The objectives of this study were: (1) to evaluate the physicochemical properties of the self-induced anaerobic fermented product (SIAFP) in comparison with those of DTFP and WTFP; (2) to evaluate their effects on growth performance and apparent total tract digestibility (ATTD) in broilers; and (3) to determine the optimal dietary inclusion level of SIAFP in broiler diets.

## 2. Materials and Methods

### 2.1. Activation and Scale-Up Culture of Probiotics

*B*. *subtilis* M6 (M6) and *L*. *plantarum* R101 (R101), stored at −20 °C, were reactivated and individually inoculated into sterilized 3% (*w*/*v*) tryptic soy broth (TSB; HiMedia, Shenzhen, China) and 5.51% (*w*/*v*) de Man, Rogosa, and Sharpe broth (MRSB; HiMedia, Shenzhen, China), respectively. The cultures were incubated aerobically at 37 °C for 24 h to obtain bacterial culture suspensions (BCS) of M6 (pH > 8.0; >8 log CFU/mL) and R101 (pH < 5.0; >9 log CFU/mL).

### 2.2. Preparation of M6 Fermentation Broth

The M6 fermentation broth was prepared by modifying the method described by Yu et al. [15]. The liquid medium contained 3% (*w*/*w*) soybean meal, 0.86% (*w*/*w*) glucose, and 1.0% (*w*/*w*) dipotassium hydrogen phosphate. After sterilization, 5% (*w*/*w*) M6 BCS was inoculated into the medium, followed by aerobic incubation at 30 °C for 48 h to obtain the M6 fermentation broth (pH > 8.0; viable count > 8.9 log CFU/mL).

### 2.3. Preparation of Fermented Products

#### 2.3.1. Wet-Form Two-Stage Fermented Product (WTFP)

Soybean meal and soybean hulls were used as the substrate at a ratio of 7:3 (*w*/*w*) without sterilization. In the first stage, 10% (*w*/*w*) M6 fermentation broth was inoculated, and the moisture content was adjusted to 55%. The mixture was aerobically fermented at 37 °C for 2 days. In the second stage, 5% (*w*/*w*) R101 BCS was added, and the moisture content was adjusted to 45%. The mixture was then sealed in composite aluminum foil bags (PET/Al/LLDPE; Chia Chyi Matrix Co., Ltd., Chiayi, Taiwan) and subjected to anaerobic fermentation at room temperature for 3 days to obtain the WTFP.

#### 2.3.2. Dry-Form Two-Stage Fermented Product (DTFP)

The WTFP was dried in a hot-air oven at 55 °C until the moisture content was reduced to below 12%, followed by grinding to obtain the DTFP.

#### 2.3.3. Self-Induced Anaerobic Fermented Product (SIAFP)

Using the same substrate, 35% (*w*/*w*) M6 fermentation broth and 5% (*w*/*w*) R101 BCS were simultaneously added, and the moisture content was adjusted to 45%. The mixture was sealed in composite aluminum foil bags (PET/Al/LLDPE) and anaerobically fermented at room temperature for 3 days to obtain the SIAFP.

### 2.4. Experimental Design and Animal Management

All experiments were conducted according to the Ross 308 nutrient requirements and management recommendations [16,17].

#### 2.4.1. Trial 1

A total of 240 day-old Ross 308 broilers (initial body weight, 36.4 ± 1.7 g; sex ratio 1:1) were randomly allocated to four dietary treatments in a randomized complete block design (RCBD) with four replicates per treatment. The treatments consisted of diets with a 2.5% inclusion of unfermented product (UFP), DTFP, WTFP, or SIAFP. The experimental period lasted 35 days. Feed and water were provided ad libitum. The composition and characteristics of the experimental diets are shown in Supplementary Materials Tables S1 and S2, including the protein and energy contents of each dietary treatment.

#### 2.4.2. Trial 2

Apparent total tract digestibility (ATTD) was determined using the difference method according to Yeh et al. [18], Moscoso-Muñoz et al. [19] and Wang et al. [20], with 0.3% (*w*/*w*) chromium oxide (Cr_2_O_3_) as an indigestible marker. A basal diet (Table S3) was mixed with each test product at a ratio of 7:3 (dry matter basis). Both the two-stage fermented product (TFP) and SIAFP were used in dried form in this trial for chemical analysis and to ensure uniform mixing with the basal diet. A total of 48 twenty-one-day-old male Ross 308 broilers (831 ± 61.3 g) were allocated to four treatments in an RCBD: basal diet, UFP, TFP, and SIAFP, with four replicates per treatment. Before the trial, birds were fasted for 12 h to empty the gastrointestinal tract. Subsequently, the experimental diets were provided for 3 consecutive days, with 50 g of feed offered every 12 h, and water was provided ad libitum. Excreta were collected daily and stored at −20 °C. Feed and excreta samples were dried at 60 °C to constant weight and ground to pass through a 1 mm sieve prior to analysis.

#### 2.4.3. Trial 3

A total of 288 day-old Ross 308 broilers (initial body weight, 35.3 ± 2.3 g; sex ratio 1:1) were used. Birds were randomly assigned to four dietary treatments in an RCBD. The treatments consisted of diets with a 0, 1.25, 2.5, or 3.75% inclusion of SIAFP, with six replicates per treatment. The experimental period lasted 35 days. The composition of the experimental diets is shown in Table S4, including the protein and energy contents of each dietary treatment.

During the storage trial in Trial 3, SIAFP was packaged into individually sealed bags. Samples were randomly collected at 0, 1, 3, 6, and 12 months for analysis, with each time point using samples from independent packages (*n* = 4).

### 2.5. Measurements and Analytical Methods

#### 2.5.1. Moisture, pH, Microbial Counts, and Protease Activity

Moisture content was determined using an infrared moisture analyzer (AND ML-50, A&D Company Limited, Tokyo, Japan). pH was measured by homogenizing the sample with distilled water at a ratio of 1:9 (*w*/*w*), followed by measurement using a benchtop pH meter (Sartorius, Göttingen, Germany). Microbial counts were determined by serial dilution of the sample in 0.9% (*w*/*v*) sterile physiological saline. The diluted samples were spread-plated onto tryptic soy agar (TSA) plates for *Bacillus* spp. or de Man, Rogosa, and Sharpe agar (MRSA) plates for *Lactiplantibacillus* spp. The plates were incubated at 37 °C for 24 h (TSA) or 48 h (MRSA), after which colonies were counted and expressed as log CFU/g. When no colonies were observed at the lowest dilution (10^−2^), the count was reported as <2.00 log CFU/g for subsequent statistical analysis. Protease activity was measured according to the methods described by Lee et al. [5]. Absorbance was measured at 420 nm. Enzyme activity was calculated using the following formula:

$$Enzyme\;activity\left(U/g\right)=\left[\frac{\left(sample\;absorbance-blank\;absorbance\right)}{\left(0.01\times 60\right)}\right]\times dilution\;factor$$

#### 2.5.2. Proximate Composition, Amino Acids, and Mineral Analysis

Feed, fermented product, and excreta samples were dried at 60 °C and ground to pass through a 1 mm sieve prior to chemical analysis. Proximate composition and amino acid analyses were performed according to the official methods of AOAC International [21]: crude protein (CP; AOAC 984.13), ether extract (EE; AOAC 920.39), crude fiber (CF; AOAC 978.10), neutral detergent fiber (NDF) and acid detergent fiber (ADF) (AOAC 2002.04 and 973.18), ash (AOAC 942.05), calcium (Ca; AOAC 968.08), phosphorus (P; AOAC 965.17), and amino acids (AOAC 994.12). Gross energy (GE) was determined using an adiabatic bomb calorimeter (LECO AC500, LECO Corp., St. Joseph, MI, USA). Chromium concentration in feed and excreta was measured by atomic absorption spectrometry (Hitachi ZA3000, Hitachi High-Tech Corporation, Tokyo, Japan) after nitric–perchloric acid digestion [22].

Derived indices were calculated on a dry matter (DM) basis using the following formulas:

Organic matter (OM, %) = 100 − ash (%).

Non-fiber carbohydrate (NFC, %) = 100 − [CP (%) + EE (%) + CF (%) + ash (%)].

Hemicellulose (%) = NDF (%) − ADF (%).

GE/OM (kcal/kg) = GE (kcal/kg)/(OM/100).

CP/GE (g/Mcal) = [CP (%) × 10]/[GE (kcal/kg)/1000].

#### 2.5.3. Growth Performance

Body weight (BW) and feed remaining were recorded weekly. The number of surviving birds was also recorded. Body weight gain (WG), feed intake (FI), feed conversion ratio (FCR = FI/WG), survival rate (SR = (final number of birds/initial number of birds) × 100), and performance efficiency factor (PEF) were calculated. The formula for PEF was as follows:

$$PEF=\frac{\left[BW\left(kg\right)\times SR\left(\%\right)\right]}{\left[FCR\times age(days)\right]}\times 100$$

#### 2.5.4. Apparent Total Tract Digestibility (ATTD) and Metabolizable Energy

The apparent total tract digestibility (ATTD) of nutrients in the basal diet (Table S5) was calculated using the following formula:

$$ATTD\left(DM\;basis,\%\right)=100\times \left[1-\left(\left(\frac{Cr_{feed}}{Cr_{excreta}}\right)\times \left(\frac{Nutrient_{excreta}}{Nutrient_{feed}}\right)\right)\right]$$

where

Cr_feed_ = chromium concentration in the diet (%),

Cr_excreta_ = chromium concentration in the excreta (%),

Nutrient_feed_ = nutrient concentration in the diet (%),

Nutrient_excreta_ = nutrient concentration in the excreta (%).

The ATTD of the fermented products (ATTD_fp_) was calculated using the difference method. The total undigested nutrient in the experimental diet was first calculated, then the contribution from the basal diet (70%) was subtracted. The remaining undigested nutrient was attributed to the fermented product (30%), and the digestibility of the fermented product was calculated using the following formula:

$$ATTD_{fp}\left(DM\;basis,\%\right)=100-\left[\frac{\left(\left(1-\frac{ATTD_{trt}}{100}\right)\times \left(0.7\times N_{b}+0.3\times N_{fp}\right)-\left(0.7\times N_{b}\times \left(1-\frac{ATTD_{b}}{100}\right)\right)\right)}{\left(0.3\times N_{fp}\right)\times 100}\right]$$

where

ATTD_b_ = ATTD of the basal diet (%),

ATTD_trt_ = ATTD of the treatment diet (%),

ATTD_fp_ = ATTD of the fermented product (%),

N_b_ = nutrient concentration in the basal diet (%),

N_fp_ = nutrient concentration in the fermented product (%).

Apparent metabolizable energy (AME) was calculated as:

$$AME\left(DM\;basis,kcal/kg\right)=GE_{feed}\times \left[1-\left(\frac{\left(\frac{Cr_{feed}}{Cr_{excreta}}\right)}{\left(\frac{GE_{excreta}}{GE_{feed}}\right)}\right)\right]$$

Nitrogen-corrected apparent metabolizable energy (AMEn) was calculated as:

$$AMEn\left(DM\;basis,kcal/kg\right)=AME-\left[8.22\times \left(\left(N_{feed}\times \left(\frac{Cr_{excreta}}{Cr_{feed}}\right)\right)-N_{excreta}\right)\right]$$

where

GE_feed_ = GE of the diet (kcal/kg),

GE_excreta_ = GE of the excreta (kcal/kg),

Cr_feed_ = chromium concentration in the diet (%),

Cr_excreta_ = chromium concentration in the excreta (%),

N_feed_ = nitrogen content in the diet (%) (calculated as CP/6.25),

N_excreta_ = nitrogen content in the excreta (%),

8.22 = nitrogen correction factor (kcal/g nitrogen).

### 2.6. Statistical Analysis

All experimental data were analyzed using SAS software (version 9.4; SAS Institute Inc., Cary, NC, USA). Prior to analysis, data were tested for normality using the Shapiro–Wilk test and for homogeneity of variances using Levene’s test. Continuous variables, including moisture, pH, microbial counts, protease activity, proximate composition, amino acid composition, ATTD, and growth performance, were analyzed using a mixed-effects model. Means were separated using the Tukey–Kramer multiple comparison test. Changes in the physicochemical properties of fermented products before and after anaerobic fermentation were analyzed using paired-sample *t*-tests. Differences among products were analyzed using independent-sample *t*-tests or mixed models, depending on the number of groups. Data from the storage trial were analyzed using a mixed-effects model, with repeated measures over time accounted for in the model. Linear and quadratic orthogonal contrasts were performed using the IML procedure to evaluate the effects of SIAFP inclusion levels. Statistical significance was declared at *p* < 0.05.

## 3. Results

### 3.1. Trial 1

#### 3.1.1. Characteristics of Fermented Products Produced by Different Processes

The characteristics of fermented products produced through different processes are presented in Table 1. During the first stage of aerobic fermentation (2 days), moisture content decreased (*p* < 0.05), while pH and *Bacillus*-like counts increased (*p* < 0.05). In the second stage of anaerobic fermentation (3 days), pH and *Bacillus*-like counts decreased, whereas *Lactiplantibacillus*-like counts increased (*p* < 0.05). Prior to anaerobic fermentation, the two-stage fermentation process showed higher pH and *Bacillus*-like counts than the SIAFP process. After fermentation, the SIAFP process exhibited the highest *Lactiplantibacillus*-like counts (*p* < 0.05). Among the final fermented products, the WTFP and SIAFP groups had higher moisture content (*p* < 0.05), while the SIAFP group showed the lowest pH (*p* < 0.05). The WTFP group had the highest *Bacillus*-like counts, whereas the SIAFP group had the highest *Lactiplantibacillus*-like counts (*p* < 0.05). Neutral protease activity (NPA) was higher in the DTFP and WTFP groups, whereas Acid protease activity (APA) was highest in the SIAFP group (*p* < 0.05). After 21 days of storage, *Lactiplantibacillus*-like counts decreased in all fermented product groups (*p* < 0.05). In the WTFP and SIAFP groups, pH, *Lactiplantibacillus*-like counts, and NPA decreased (*p* < 0.05), whereas APA increased (*p* < 0.05).

#### 3.1.2. Growth Performance

The effects of fermented products derived from different processes on growth performance in broilers are presented in Table 2. During the 21–35 and 0–35 day periods, broilers fed DTFP, WTFP, and SIAFP exhibited significantly higher weight gain (WG) and performance efficiency factor (PEF) than those fed the UFP diet (*p* < 0.05).

### 3.2. Trial 2

#### 3.2.1. Proximate Composition of Fermented Products

The proximate composition of fermented products derived from different processes is presented in Table 3. Dry matter (DM) recovery and organic matter (OM) content were highest in the UFP group, followed by the SIAFP group, and lowest in the TFP group (*p* < 0.05). In contrast, ash content showed the opposite trend (*p* < 0.05). The SIAFP group had the highest crude protein (CP) content (*p* < 0.05), whereas ether extract (EE) and non-fiber carbohydrates (NFC) contents were highest in the UFP group (*p* < 0.05). Crude fiber (CF) content was highest in the TFP group (*p* < 0.05), while neutral detergent fiber (NDF) content was lowest in the UFP group (*p* < 0.05). Acid detergent fiber (ADF), calcium (Ca), and phosphorus (P) contents were highest in the TFP group, followed by the SIAFP group, and lowest in the UFP group (*p* < 0.05).

#### 3.2.2. Amino Acid Composition of Fermented Products

The amino acid composition of fermented products derived from different processes is presented in Table 4. Among the essential amino acids, Arg, His, and Lys contents were higher in the TFP and SIAFP groups than in the UFP group (*p* < 0.05). Leu content was higher in the SIAFP group than in the TFP group (*p* < 0.05), and Met content was highest in the SIAFP group (*p* < 0.05). Thr content was higher in the SIAFP group than in the UFP group, with the TFP group being intermediate (*p* < 0.05). For non-essential amino acids, Cys and Ser contents were higher in the TFP and SIAFP groups than in the UFP group (*p* < 0.05). Total essential amino acid and total amino acid contents followed the order SIAFP > TFP > UFP (*p* < 0.05). In addition, total non-essential amino acid content was higher in the SIAFP group than in the TFP and UFP groups (*p* < 0.05).

#### 3.2.3. Apparent Total Tract Digestibility of Fermented Products in Broilers

The apparent total tract digestibility (ATTD) of fermented products produced by different processes in broilers is presented in Table 5. The ATTD of CF was higher in the TFP group than in the SIAFP and UFP groups (*p* < 0.05). The ATTD of ADF and Ca was higher in the TFP group than in the UFP group (*p* < 0.05). The ATTD of hemicellulose was higher in the SIAFP group than in the UFP group (*p* < 0.05).

### 3.3. Trial 3

#### 3.3.1. Characteristics of SIAFP During Storage

The characteristics of SIAFP during storage are presented in Table 6. The pH of SIAFP decreased from month 0 to month 1 and remained stable thereafter (*p* < 0.05; linear, *p* < 0.05; quadratic, *p* < 0.05). *Lactiplantibacillus*-like counts decreased with increasing storage duration, reaching the lowest level at month 6 and remaining stable thereafter (*p* < 0.05; linear, *p* < 0.05; quadratic, *p* < 0.05).

#### 3.3.2. Growth Performance

The effects of different dietary levels of SIAFP on growth performance in broilers are presented in Table 7. WG and feed conversion ratio (FCR) during the 21–35 and 0–35 day periods, as well as PEF during 0–35 days, showed linear or quadratic responses to increasing dietary SIAFP inclusion levels (*p* < 0.05; linear, *p* < 0.05; quadratic, *p* < 0.05).

## 4. Discussion

The changes in physicochemical properties and microbial counts reflected differences in microbial dynamics among the various fermentation processes. During the aerobic stage of the two-stage fermentation process, moisture content decreased, while pH and *Bacillus*-like counts increased, mainly due to metabolic heat production, enhanced evaporation by aeration, and water consumption by microbial metabolism [23,24,25] and rapid proliferation of M6, accompanied by ammonia accumulation [26,27]. Upon transition to anaerobic conditions, pH and *Bacillus*-like counts declined, whereas *Lactiplantibacillus*-like counts increased, indicating microbial succession toward R101 dominance [28,29,30].

SIAFP exhibited the lowest final pH and highest *Lactiplantibacillus*-like counts, suggesting that the self-induced anaerobic process created a more favorable ecological niche for R101 proliferation with reduced competition [31,32,33]. In contrast, the initial aerobic phase in TFP helped maintain higher *Bacillus*-like counts and buffered acidification. In the final products, WTFP and SIAFP retained higher microbial counts than DTFP, primarily due to reduced thermal and mechanical stresses induced by hot-air drying and grinding [3,18,34]. Higher NPA in DTFP and WTFP was associated with M6 activity during aerobic fermentation, whereas elevated APA in SIAFP reflected R101 predominance under anaerobic conditions.

After 21 days of storage, *Bacillus*-like counts remained stable in all groups (*p* > 0.05), indicating strong storage stability of M6. In contrast, *Lactiplantibacillus*-like counts and NPA decreased, whereas APA increased in WTFP and SIAFP during storage, suggesting that the metabolic activity of R101 persisted but was gradually restricted by nutrient depletion and metabolite accumulation [35,36,37]. These differential changes in microbial counts and enzyme activities indicate a shift in microbial metabolic status during storage, in which M6 likely entered dormancy, limiting NPA production, while R101 maintained relatively higher APA under acidic conditions [38,39]. Collectively, these findings suggest that self-induced anaerobic fermentation effectively modulates microbial dynamics and enzymatic activity, thereby enhancing product stability and functionality.

From a nutritional perspective, all fermented product supplementation groups improved WG and PEF in broilers compared with the UFP group, confirming the growth-promoting effects of fermented feed products. Consistent with our previous studies on two-stage fermented products (DTFP and WTFP) using soybean meal and soybean hulls inoculated with M6 and R101 [7,8,9]. DTFP and WTFP fermented feeds have been shown to enhance broiler performance when included in diets. Our study further shows that SIAFP also has growth-promoting benefits, which are generally associated with the degradation of anti-nutritional factors and the production of probiotics, organic acids, and other functional metabolites during fermentation [1,2,3].

Despite differences in processing conditions, WTFP and DTFP showed comparable growth performance (*p* > 0.05). Although DTFP contained higher nutrient concentrations due to lower moisture content, WTFP likely maintained similar efficacy through better preservation of microbial viability and protease activity in the absence of drying stress [6]. Similarly, SIAFP achieved growth performance comparable to that of DTFP and WTFP despite lacking a distinct aerobic fermentation stage. The comparable microbial counts and enzyme activities observed in the diets suggest that sufficient functional metabolites were generated to support broiler growth (Table S2). In addition, the self-induced anaerobic fermentation process simplifies production by reducing processing time, equipment requirements, and energy consumption, highlighting its potential as a practical and cost-effective alternative for fermented feed production.

Fermentation-induced compositional changes further elucidate nutrient transformation mechanisms. The lower DM recovery and OM contents in the TFP and SIAFP groups compared with the UFP group indicate substantial OM loss during fermentation, likely due to microbial metabolism and volatilization of gaseous compounds [40,41]. Notably, OM loss was lower in SIAFP than in TFP, suggesting that sealed anaerobic fermentation reduced volatilization losses [29,42,43].

Carbohydrate and lipid contents decreased after fermentation, reflecting microbial utilization of these substrates for the production of organic acids and other metabolites [44,45,46]. In contrast, CP and total amino acid contents increased after fermentation, following the ranking SIAFP > TFP > UFP. The self-induced anaerobic fermentation process was dominated by R101, followed by M6. Under anaerobic conditions, these microorganisms retain the capacity for amino acid synthesis, while most amino acids are less susceptible to extensive degradation [28,47,48]. Consequently, the greater CP and amino acid accumulation observed in SIAFP likely reflected improved nitrogen retention under anaerobic conditions, whereas the aerobic stage in TFP may have promoted partial nitrogen loss through ammonia volatilization [49,50,51]. These results indicate that self-induced anaerobic fermentation is more favorable for CP and amino acid preservation and accumulation.

Fiber-related components (CF, NDF, and ADF) increased after fermentation, mainly due to preferential utilization of soluble carbohydrates and lipids during microbial metabolism [52,53]. The increase was greater in TFP than in SIAFP, consistent with the higher OM loss observed in TFP.

Mineral contents (Ca and P) followed a similar trend to ash, indicating that minerals remained relatively stable during fermentation and were not significantly lost through microbial metabolism. This observation is consistent with the chemical stability of minerals [54]. Furthermore, there were no significant differences in GE or GE/OM ratio among the groups (*p* > 0.05), suggesting that fermentation primarily altered nutrient distribution rather than total energy content.

Digestibility results further emphasize the functional benefits of fermentation. The two-stage fermentation process improved the ATTD of CF and ADF, with a tendency to increase NDF and hemicellulose digestibility (*p* < 0.1). These improvements likely reflect enhanced fiber pre-degradation during fermentation, as broilers have limited endogenous capacity to digest structural carbohydrates [55].

During the aerobic stage, *B. subtilis* secretes cellulases and hemicellulases that disrupt fiber structure [56,57,58], promoting initial fiber degradation. In the subsequent anaerobic stage, *L. plantarum* further contributes to polysaccharide breakdown via enzymes such as endoglucanases, exoglucanases, β-glucosidases, and mannanases [59]. These sequential microbial activities likely explain the greater CF degradation and improved ATTD of most fiber fractions in the TFP group. In contrast, SIAFP, which lacks a distinct aerobic phase, showed reduced CF degradation, while NDF and ADF degradation remained comparable (*p* > 0.05), suggesting that secondary enzymatic activity under anaerobic conditions is sufficient to maintain partial fiber conversion, albeit with reduced efficiency at the level of bulk fiber fractions.

Beyond fiber degradation, it biochemically transforms complex carbohydrates into more readily utilizable forms, thereby enhancing overall nutrient utilization efficiency. As indicated by higher ATTD of OM and NFC in the TFP group (*p* < 0.1), suggesting enhanced substrate availability through microbial pre-digestion.

The ATTD of Ca was higher in TFP than in UFP, likely due to microbial degradation of phytate complexes that enhances mineral availability [60,61,62]. Although *L. plantarum* also contributes to phytate degradation [63,64], its efficiency appears lower [65], which may explain the slightly reduced Ca digestibility in SIAFP. However, Ca digestibility can be influenced by endogenous Ca excretion [66]; therefore, the present results should be interpreted as indicators of overall Ca absorption rather than true digestibility.

Overall, these findings suggest that fermentation plays a crucial role in coordinating enzymatic reactions and the bioavailability of downstream nutrients. The TFP system confers superior digestibility of most fiber components, whereas the SIAFP system retains selective advantages in hemicellulose digestibility.

Long-term storage (12-month) results demonstrate that *Bacillus*-like counts remained stable in SIAFP (*p* > 0.05), indicating good storage stability of *B. subtilis* in SIAFP. The pH decreased during the early storage stage and then stabilized at an acidic level after one month, suggesting microbial metabolic activity persisted initially, leading to continued production of organic acids. As acidity increased, microbial activity was progressively inhibited, and the system transitioned into a dynamic equilibrium state [67,68,69].

In contrast, *L. plantarum* exhibits a reduced long-term survival rate compared to *B. subtilis* due to its non-spore-forming nature [30]. Although *Lactiplantibacillus*-like counts in SIAFP gradually decreased and stabilized at lower levels after 6 months, the fermentation system successfully maintained a persistent acidic microenvironment and stable *Bacillus* populations. These findings underscore the robust practical application potential of SIAFP, as its stability throughout extended storage duration facilitates efficient feed preservation and logistics. These results indicate that SIAFP has strong potential for practical application, as its stability during long-term storage is advantageous for feed preservation and transportation.

Dietary supplementation with 1.25–3.75% SIAFP improved broiler growth performance during both the 21–35 d and 0–35 d periods, with the best responses observed at 1.25% and 2.5% inclusion levels, indicating a non-linear dose effect.

The growth-promoting effects of SIAFP may be associated with increased dietary *Bacillus*-like and *Lactiplantibacillus*-like populations, as well as improved nutrient composition and digestibility, including higher CP, amino acids, and improved ATTD of ADF, hemicellulose, and Ca (Table 4, Table 5 and Table S4), which collectively enhance nutrient utilization efficiency. However, the growth response plateaued or slightly declined at higher inclusion levels, suggesting an optimal supplementation range rather than a dose-dependent linear effect.

In summary, the self-induced anaerobic fermentation technology developed in this study provides a practical and cost-effective alternative to traditional two-stage fermentation, while achieving comparable growth-promoting effects in broilers. However, several limitations should be acknowledged. Trials 1 and 2 were conducted with four replicates per treatment and were primarily designed as exploratory evaluations. Consequently, the statistical power to detect subtle treatment differences may have been limited, which contributed to the large variation observed in some parameters. Some non-significant results should therefore be interpreted with caution. Nevertheless, consistent trends were observed across multiple parameters, supporting the overall conclusions of the study.

In addition, although the freshly prepared SIAFP demonstrated significant growth-promoting effects in broilers, the storage trial revealed a significant decline in *Lactiplantibacillus*-like counts after one month. Whether long-term stored SIAFP can maintain comparable biological efficacy and growth-promoting effects remains to be determined. Future studies with larger sample sizes are warranted to further verify the stability and practical applicability of SIAFP under commercial conditions.

Overall, the results suggest that dietary inclusion of 1.25–2.5% SIAFP is sufficient to effectively enhance growth performance in broilers.

## 5. Conclusions

The one-stage self-induced anaerobic fermented product (SIAFP), as well as the dry- and wet-form two-stage fermented products, improved the growth performance of broiler chickens. Compared with the two-stage fermentation products, SIAFP demonstrated greater potential for practical application due to its simpler processing and reduced production time. Dietary inclusion of 1.25–2.5% SIAFP effectively enhanced growth performance, which was associated with increased crude protein and total amino acid contents and improved apparent total tract digestibility.

## Figures and Tables

**Table 1:** Characteristics of fermented products produced by different processes.

Stage of Fermentation	UFP ^1^	DTFP ^1^	WTFP ^1^	SIAFP ^1^	SEM	*p*-Value
	Moisture (%)					
Aerobic day 0	-	55.0 ^x^		-	-	-
Aerobic day 2	-	42.1 ^y^		-	-	-
SEM	-	2.7				
*p*-Value	-	0.033				
Anaerobic day 0	-	44.9		44.8	2.6	0.378
Anaerobic day 3	-	44.8		45.0	0.4	0.243
SEM	-	2.6		0.1		
*p*-Value	-	0.331		0.219		
Final product	7.7	9.7	44.8	44.5	-	-
Storage day 21	7.7	9.8	44.8	44.7	-	-
SEM	<0.1	0.1	0.4	0.1		
*p*-Value	0.600	0.401	0.499	0.127		
	pH					
Aerobic day 0	-	6.1 ^y^		-	-	-
Aerobic day 2	-	8.4 ^x^		-	-	-
SEM	-	<0.1		-		
*p*-Value	-	<0.001		-		
Anaerobic day 0	-	8.3 ^a,x^		6.4 ^b,x^	<0.1	<0.001
Anaerobic day 3	-	6.4 ^a,y^		4.5 ^b,y^	<0.1	<0.001
SEM	-	<0.1		<0.1		
*p*-Value	-	<0.001		<0.001		
Final product	6.1 ^c^	6.3 ^b^	6.4 ^a,x^	4.5 ^d,x^	<0.1	<0.001
Storage day 21	6.1 ^c^	6.3 ^a^	6.2 ^b,y^	4.2 ^d,y^	<0.1	<0.001
SEM	<0.1	<0.1	<0.1	<0.1		
*p*-Value	0.905	0.703	0.001	<0.001		
	*Bacillus*-like (log CFU/g)					
Aerobic day 0	-	7.60 ^y^		-	-	-
Aerobic day 2	-	9.37 ^x^		-	-	-
SEM	-	0.02		-		
*p*-Value	-	<0.001		-		
Anaerobic day 0	-	9.34 ^a,x^		8.26 ^b^	0.02	<0.001
Anaerobic day 3	-	8.85 ^a,y^		8.27 ^b^	0.04	<0.001
SEM	-	0.02		0.02		
*p*-Value	-	<0.001		0.690		
Final product	<2.0 ^d^	8.56 ^b^	8.85 ^a^	8.27 ^c^	0.02	<0.001
Storage day 21	<2.0 ^d^	8.55 ^b^	8.84 ^a^	8.26 ^c^	0.02	<0.001
SEM	-	0.03	0.01	0.02		
*p*-Value	-	0.706	0.375	0.527		
	*Lactiplantibacillus*-like (log CFU/g)					
Anaerobic day 0	-	7.43 ^y^		7.45 ^y^	0.03	0.595
Anaerobic day 3	-	8.44 ^b,x^		9.53 ^a,x^	0.03	<0.001
SEM	-	0.04		0.02		
*p*-Value	-	0.001		<0.001		
Final product	<2.0 ^d^	5.56 ^c,x^	8.44 ^b,x^	9.53 ^a,x^	0.03	<0.001
Storage day 21	<2.0 ^d^	5.33 ^c,y^	7.33 ^b,y^	8.75 ^a,y^	0.04	<0.001
SEM	-	0.05	0.02	0.04		
*p*-Value	-	0.020	<0.001	<0.001		
	Neutral protease activity (U/g)					
Final product	N.D. ^c^	761 ^a^	814 ^a,x^	401 ^b,x^	12.7	<0.001
Storage day 21	N.D. ^d^	742 ^a^	626 ^b,y^	205 ^c,y^	8.3	<0.001
SEM	-	19.7	27.4	17.0		
*p*-Value	-	0.400	0.006	0.001		
	Acid protease activity (U/g)					
Final product	N.D. ^c^	42.5 ^b^	45.3 ^b,y^	312 ^a,y^	16.2	<0.001
Storage day 21	N.D. ^c^	42.3 ^b^	54.5 ^b,x^	409 ^a,x^	13.1	<0.001
SEM	-	5.1	1.5	21.1		
*p*-Value	-	0.964	0.009	0.020		

*n* = 4. ^a–d^ Means in the same row with different superscripts are significantly different (*p* < 0.05). ^x,y^ Means in the same column with different superscripts are significantly different (*p* < 0.05). ^1^ UFP, unfermented product; DTFP, dry-form two-stage fermented product; WTFP, wet-form two-stage fermented product; SIAFP, self-induced anaerobic fermented product. N.D. = not detected.

**Table 2:** Effects of fermented products produced by different processes on growth performance in broilers.

Period (d)	UFP ^1^	DTFP ^1^	WTFP ^1^	SIAFP ^1^	SEM	*p*-Value
	Feed intake, g/bird					
0–21	1033	1044	1043	1040	20	0.976
21–35	1965	2013	1992	1988	37	0.840
0–35	2998	3057	3035	3028	44	0.820
	Weight gain, g/bird					
0–21	806	816	832	820	12	0.564
21–35	1186 ^b^	1282 ^a^	1265 ^a^	1268 ^a^	17	0.014
0–35	1991 ^b^	2098 ^a^	2096 ^a^	2087 ^a^	20	0.011
	Feed conversion ratio ^2^					
0–21	1.28	1.28	1.26	1.27	0.01	0.345
21–35	1.66	1.57	1.58	1.57	0.03	0.166
0–35	1.51	1.46	1.45	1.45	0.02	0.119
	Survival, %					
0–35	100	100	100	100	-	-
	Performance efficiency factor ^3^					
0–21	313	317	329	321	4	0.138
0–35	385 ^b^	419 ^a^	421 ^a^	419 ^a^	7	0.010

*n* = 4. ^a,b^ Means in the same row with different superscripts are significantly different (*p* < 0.05). ^1^ UFP, unfermented product; DTFP, dry-form two-stage fermented product; WTFP, wet-form two-stage fermented product; SIAFP, self-induced anaerobic fermented product. ^2^ Feed conversion ratio = feed intake/weight gain. ^3^ Performance efficiency factor = [(body weight (kg) × survival (%))/(feed conversion ratio × age (days))] × 100.

**Table 3:** Proximate analysis of fermented products produced by different processes.

Items (DM Basis)	UFP ^1^	TFP ^1^	SIAFP ^1^	SEM	*p*-Value
Recovery yield (%)	100.0 ^a^	78.0 ^c^	91.1 ^b^	0.2	<0.001
Organic matter (%)	93.2 ^a^	91.3 ^c^	92.5 ^b^	<0.1	<0.001
Ash (%)	6.83 ^c^	8.75 ^a^	7.52 ^b^	0.14	<0.001
Gross energy (kcal/kg)	4205	4353	4304	80	0.463
GE/OM ^2^ (kcal/kg)	4514	4770	4654	86	0.187
Crude protein (%)	38.1 ^b^	38.8 ^b^	42.4 ^a^	0.8	0.017
CP/GE ^3^ (g/Mcal)	90.7	89.3	98.6	2.4	0.066
Ether extract (%)	0.88 ^a^	0.76 ^b^	0.71 ^b^	0.01	<0.001
Non-fiber carbohydrates (%)	32.5 ^a^	24.1 ^b^	22.6 ^b^	1.2	0.002
Crude fiber (%)	13.4 ^b^	17.8 ^a^	13.1 ^b^	0.2	<0.001
Neutral detergent fiber (%)	21.7 ^b^	27.6 ^a^	26.4 ^a^	0.5	0.001
Acid detergent fiber (%)	17.4 ^c^	24.0 ^a^	21.4 ^b^	0.4	<0.001
Hemicellulose (%)	4.26	3.61	4.98	0.56	0.293
Calcium (%)	0.48 ^c^	0.65 ^a^	0.56 ^b^	0.01	0.001
Phosphorus (%)	0.55 ^c^	0.70 ^a^	0.61 ^b^	0.01	0.001

*n* = 4. ^a–c^ Means in the same row with different superscripts are significantly different (*p* < 0.05). ^1^ UFP, unfermented product; TFP, two-stage fermented product; SIAFP, self-induced anaerobic fermented product. ^2^ Gross energy per organic matter. ^3^ Crude protein per gross energy.

**Table 4:** Amino acid composition of fermented products produced by different processes.

Items (DM Basis, %)	UFP ^1^	TFP ^1^	SIAFP ^1^	SEM	*p*-Value
Recovery yield	100.0 ^a^	78.0 ^c^	91.1 ^b^	0.2	<0.001
Essential amino acid					
Arg	1.73 ^b^	2.34 ^a^	2.52 ^a^	0.05	<0.001
His	0.69 ^b^	0.87 ^a^	0.93 ^a^	0.02	0.001
Ile	1.62	1.58	1.69	0.06	0.396
Leu	2.91 ^ab^	2.80 ^b^	3.03 ^a^	0.05	0.046
Lys	1.51 ^b^	2.14 ^a^	2.18 ^a^	0.06	0.001
Met	0.29 ^b^	0.29 ^b^	0.38 ^a^	0.01	<0.001
Phe	1.85	1.78	1.94	0.05	0.172
Thr	1.24 ^b^	1.38 ^ab^	1.49 ^a^	0.04	0.011
Val	1.87	1.74	1.87	0.07	0.414
Non-essential amino acid					
Ala	1.69	1.54	1.65	0.06	0.222
Asp	4.05	3.92	4.27	0.15	0.316
Cys	0.22 ^b^	0.31 ^a^	0.33 ^a^	0.01	0.001
Glu	5.74	6.09	6.56	0.20	0.073
Gly	1.67	1.65	1.74	0.04	0.237
Pro	1.82	1.85	2.11	0.08	0.077
Ser	1.49 ^b^	1.74 ^a^	1.85 ^a^	0.05	0.008
Tyr	1.04	1.02	1.13	0.04	0.228
Total essential amino acids	13.7 ^c^	14.9 ^b^	16.0 ^a^	0.1	<0.001
Total non-essential amino acids	17.7 ^b^	18.1 ^b^	19.6 ^a^	0.2	0.003
Total amino acids	31.4 ^c^	33.0 ^b^	35.7 ^a^	0.2	<0.001

*n* = 4. ^a–c^ Means in the same row with different superscripts are significantly different (*p* < 0.05). ^1^ UFP, unfermented product; TFP, two-stage fermented product; SIAFP, self-induced anaerobic fermented product.

**Table 5:** Apparent total tract digestibility of fermented products produced by different processes in broilers.

Items (DM Basis)	UFP ^1^	TFP ^1^	SIAFP ^1^	SEM	*p*-Value
Organic matter (%)	44.6	57.8	46.7	3.1	0.057
Ash (%)	33.0	49.4	35.8	6.9	0.272
AMEn ^2^ (kcal/kg)	3363	3723	3499	100	0.109
Crude protein (%)	53.8	51.1	56.5	4.6	0.728
Ether extract (%)	58.4	77.8	75.0	20.4	0.778
Non-fiber carbohydrates (%)	57.0	81.4	41.0	9.7	0.066
Crude fiber (%)	2.2 ^b^	19.4 ^a^	0.1 ^b^	2.7	0.004
Neutral detergent fiber (%)	10.5	42.9	36.0	8.9	0.092
Acid detergent fiber (%)	7.7 ^b^	31.1 ^a^	15.3 ^ab^	4.9	0.039
Hemicellulose (%)	14.7 ^b^	66.3 ^ab^	79.2 ^a^	13.9	0.036
Calcium (%)	17.7 ^b^	55.7 ^a^	37.7 ^ab^	5.1	0.006
Phosphorus (%)	53.0	60.8	42.8	9.3	0.442

*n* = 4. ^a,b^ Means in the same row with different superscripts are significantly different (*p* < 0.05). ^1^ UFP, unfermented product; TFP, two-stage fermented product; SIAFP, self-induced anaerobic fermented product. ^2^ Apparent metabolizable energy corrected for nitrogen.

**Table 6:** Characteristics of SIAFP during storage.

Items	Storage Period (Months)					SEM	*p*-Value	Orthogonal Contrasts	
	0	1	3	6	12			Linear	Quadratic
Moisture (%)	45.0	44.7	44.8	45.1	44.9	0.5	0.977	0.808	0.851
pH	4.5 ^a^	4.2 ^b^	4.3 ^b^	4.3 ^b^	4.3 ^b^	<0.1	<0.001	0.045	0.011
*Bacillus*-like (log CFU/g)	8.27	8.26	8.27	8.25	8.18	0.20	0.997	0.729	0.905
*Lactiplantibacillus*-like (log CFU/g)	9.53 ^a^	8.75 ^b^	4.32 ^c^	2.00 ^d^	<2.00 ^d^	0.07	<0.001	<0.001	<0.001

*n* = 4. ^a–d^ Means in the same row with different superscripts are significantly different (*p* < 0.05).

**Table 7:** Effects of different dietary levels of SIAFP on growth performance in broilers.

Period (d)	SIAFP ^1^ (%)				SEM	*p*-Value	Orthogonal Contrasts	
	0	1.25	2.5	3.75			Linear	Quadratic
	Feed intake (g/bird)							
0–21	1012	1033	1021	1062	26	0.423	0.179	0.644
21–35	1021	1065	1058	1049	74	0.593	0.333	0.082
0–35	2930	3055	3019	3038	93	0.285	0.192	0.279
	Weight gain (g/bird)							
0–21	782	814	799	827.3	25	0.308	0.132	0.894
21–35	542 ^b^	601 ^a^	602 ^a^	573 ^ab^	43	0.005	0.065	0.001
0–35	1947 ^b^	2092 ^a^	2085 ^a^	2055 ^ab^	61	0.011	0.028	0.009
	Feed conversion ratio ^2^							
0–21	1.3	1.27	1.28	1.29	0.01	0.317	0.612	0.111
21–35	1.89 ^a^	1.77 ^ab^	1.76 ^b^	1.83 ^ab^	0.02	0.047	0.143	0.016
0–35	1.51 ^a^	1.46 ^ab^	1.45 ^b^	1.48 ^ab^	0.01	0.022	0.097	0.007
	Survival (%)							
0–35	100	100	100	100	-	-	-	-
	Performance efficiency factor ^3^							
0–21	301	319	311	320	12	0.216	0.129	0.498
0–35	376 ^b^	416 ^a^	419 ^a^	404 ^ab^	12	0.002	0.014	0.001

*n* = 6. ^a,b^ Means in the same row with different superscripts are significantly different (*p* < 0.05). ^1^ Self-induced anaerobic fermented product. ^2^ Feed conversion ratio = Feed intake/Weight gain. ^3^ Performance efficiency factor = [(body weight (kg) × survival (%))/(feed conversion ratio × age (days))] × 100.

## Data Availability

The data presented in this study are available on request from the corresponding author.

## Supplementary Materials

The following supporting information can be downloaded at: https://www.mdpi.com/article/10.3390/ani16121754/s1, Table S1: Ingredient composition of diets in Trial 1; Table S2: Characteristics of basal diets with trial 1; Table S3: Ingredient composition of the basal diet in Trial 2; Table S4: Ingredient composition of diets in Trial 3; Table S5: Analyzed composition and apparent digestibility of the basal diet in Trial 2.
