# Supplementary material for: Self-Induced Anaerobic Fermented Products of Bacillus subtilis M6 and Lactiplantibacillus plantarum R101 Improve Growth Performance in Broilers
Source: Animals (Basel). 2026 Jun 6;16(12):1754. doi: 10.3390/ani16121754 (PMC13295642; doi:10.3390/ani16121754)
Supplement: Supplementary file 1 [file animals-16-01754-s001.zip › animals-4337357-supplementary.pdf]

Table S1. Ingredient composition of diets in Trial 1.

Ingredients (%)	0-21 d		21-35 d	
	DP[1]	WP[1]	DP[1]	WP[1]
Corn meal	47.25	45.73	50.64	49.1
Full fat soybean meal (CP, 38%)	30.56	36.03	29.15	34.62
Soybean oil			1.10	1.10
Soybean meal (CP, 44%)	15.62	11.68	12.97	9.07
Dried product (UFP/DTFP)[2]	2.50		2.50	
Wet product (WTFP/SIAFP)[2]		2.50		2.50
Monocalcium phosphate (P, 21%)	1.47	1.46	1.34	1.34
Limestone, pulverized	1.56	1.56	1.36	1.36
Salt	0.30	0.30	0.30	0.30
DL-Methionine (DL-Met, 98%)	0.38	0.38	0.32	0.32
L-Lysine HCl (L-Lys, 78%)	0.17	0.16	0.11	0.09
Vitamin premix[3]	0.10	0.10	0.10	0.10
Mineral premix[4]	0.10	0.10	0.10	0.10
Total	100.00	100.00	100.00	100.00
Calculated value				
Crude protein (%)	23.0	23.0	21.5	21.5
Metabolic energy (kcal/kg)	3100	3100	3200	3200
Calcium (%)	0.96	0.96	0.87	0.87
Available phosphate (%)	0.48	0.48	0.44	0.44
d-Methionine + d-Cystine (%)	0.95	0.95	0.87	0.87
d-Lysine (%)	1.28	1.28	1.15	1.15

[1] DP, dried product; WP, wet product. [2] UFP, unfermented product; DTFP, dried two-stage fermented product; WTFP, wet two-stage fermented product; SIAFP, self-induced anaerobic fermented product. [3] Vitamin premix per kilogram: Vitamin A, 14,000,000 IU; Vitamin D_3, 1,200,000 IU; Vitamin E, 44,000 IU; Vitamin K_3, 2.4 g; Vitamin B_1, 3 g; Vitamin B_2, 7 g; Vitamin B_6, 3 g; Vitamin B_{12}, 0.04 g; pantothenate, 15 g; Niacin, 27 g; Biotin, 0.2 g; Folic acid, 2 g. [4] Mineral premix per kilogram: Cu ($CuSO_4{\cdot}5H_2O$), 15 g; Fe ($FeSO_4{\cdot}7H_2O$), 150 g; Mn ($MnSO_4{\cdot}H_2O$), 50 g; Zn (ZnO), 50 g; I (KI), 2 g; Se ($NaSeO_3$), 0.2 g.

Table S2. Characteristics of basal diets with trial 1.

Items	UFP[1]	DTFP[1]	WTFP[1]	SIAFP[1]	SEM	*p*-Value
			0-21 d			
Moisture, %	10.0[b]	10.1[b]	10.7[a]	10.8[a]	0.1	<0.001
Crude protein (%)	22.9	23.0	23.1	23.0	0.1	0.682
Bacillus-like, log CFU/g	2.35[d]	6.88[b]	7.44[a]	6.63[c]	0.03	<0.001
Lactiplantibacillus-like, log CFU/g	<2.00[d]	4.00[c]	6.14[b]	6.44[a]	0.06	<0.001
NPA[2] (U/g)	N.D.[c]	19.1[a]	20.4[a]	10.0[b]	0.3	<0.001
APA[2] (U/g)	N.D.[c]	1.1[b]	1.1[b]	7.8[a]	0.4	<0.001
			21-35 d			
Moisture, %	10.1[b]	10.1[b]	10.7[a]	10.8[a]	<0.1	<0.001
Crude protein (%)	21.5	21.5	21.4	21.5	0.2	0.968
Bacillus-like, log CFU/g	2.33[d]	6.80[b]	7.46[a]	6.63[c]	0.04	<0.001
Lactiplantibacillus-like, log CFU/g	<2.00[d]	4.00[c]	6.08[b]	6.38[a]	0.06	<0.001
NPA[2] (U/g)	N.D.[c]	18.6[a]	15.7[a]	5.1[b]	0.2	<0.001
APA[2] (U/g)	N.D.[c]	1.1[b]	1.4[b]	10.2[a]	0.3	<0.001

n= 4. [a-d] Means in the same row with different superscripts are significantly different ($P < 0.05$). [1] UFP, unfermented product; DTFP, dried two-stage fermented product; WTFP, wet two-stage fermented product; SIAFP, self-induced anaerobic fermented product. [2] NPA, neutral protease activity; APA, acid protease activity. N.D. = not detected

Table S3. Ingredient composition of the basal diet in Trial 2.

**Ingredients (%)**	**Basal diet**
Corn meal	50.59
Full fat soybean meal (CP, 38%)	33.93
Soybean oil	1.01
Soybean meal (CP, 44%)	10.77
Monocalcium phosphate (P, 21%)	1.35
Limestone, pulverized	1.36
Salt	0.30
DL-Methionine (DL-Met, 98%)	0.31
L-Lysine HCl (L-Lys, 78%)	0.09
Vitamin premix[1]	0.10
Mineral premix[2]	0.10
Total	100.00
Calculated value	
Crude protein (%)	21.5
Metabolic energy (kcal/kg)	3200
Calcium (%)	0.87
Available phosphate (%)	0.44
d-Methionine + d-Cystine (%)	0.87
d-Lysine (%)	1.15

[1] Vitamin premix per kilogram: Vitamin A, 14,000,000 IU; Vitamin D3, 1,200,000 IU; Vitamin E, 44,000 IU; Vitamin K3, 2.4 g; Vitamin B1, 3 g; Vitamin B2, 7 g; Vitamin B6, 3 g; Vitamin B12, 0.04 g; pantothenate, 15 g; Niacin, 27 g; Biotin, 0.2 g; Folic acid, 2 g. [2] Mineral premix per kilogram: Cu (CuSO4·5H2O), 15 g; Fe (FeSO4·7H2O), 150 g; Mn (MnSO4·H2O), 50 g; Zn (ZnO), 50 g; I (KI), 2 g; Se (NaSeO3), 0.2 g.

Table S4. Ingredient composition of diets in Trial 3.

Ingredients (%)	0-21 d SIAFP (%)				21-35 d SIAFP (%)			
	0.00	1.25	2.50	3.75	0.00	1.25	2.50	3.75
Corn meal	47.19	46.47	45.73	45.00	50.59	49.85	49.10	48.38
Full fat soybean meal (CP, 38%)	35.33	35.69	36.03	36.39	33.93	34.28	34.62	35.00
Soybean oil					1.10	1.10	1.10	1.09
Soybean meal (CP, 44%)	13.42	12.52	11.68	10.78	10.77	9.92	9.07	8.15
SIAFP[1]	0.00	1.25	2.50	3.75	0.00	1.25	2.50	3.75
Monocalcium phosphate (P, 21%)	1.48	1.47	1.46	1.45	1.35	1.35	1.34	1.33
Limestone, pulverized	1.55	1.56	1.56	1.56	1.36	1.36	1.36	1.37
Salt	0.30	0.30	0.30	0.30	0.30	0.30	0.30	0.30
DL-Methionine (DL-Met, 98%)	0.37	0.38	0.38	0.39	0.31	0.32	0.32	0.33
L-Lysine HCl (L-Lys, 78%)	0.15	0.16	0.16	0.17	0.09	0.09	0.09	0.10
Vitamin premix[2]	0.10	0.10	0.10	0.10	0.10	0.10	0.10	0.10
Mineral premix[3]	0.10	0.10	0.10	0.10	0.10	0.10	0.10	0.10
Total	100.00	100.00	100.00	100.00	100.00	100.00	100.00	100.00
Calculated value								
Crude protein (%)	23.0	23.0	23.0	23.0	21.5	21.5	21.5	21.5
Metabolic energy (kcal/kg)	3100	3100	3100	3100	3200	3200	3200	3200
Calcium (%)	0.96	0.96	0.96	0.96	0.87	0.87	0.87	0.87
Available phosphate (%)	0.48	0.48	0.48	0.48	0.44	0.44	0.44	0.44
d-Methionine + d-Cystine (%)	0.95	0.95	0.95	0.95	0.87	0.87	0.87	0.87
d-Lysine (%)	1.28	1.28	1.28	1.28	1.15	1.15	1.15	1.15
Analyzed value*								
Moisture, %	10.0	10.4	10.8	11.3	10.0	10.3	10.9	11.2
Crude protein (%)	22.9	23.0	23.1	23.2	23.1	22.9	23.2	23.0
Bacillus-like, log CFU/g	2.28	6.40	6.69	6.87	2.27	6.41	6.70	6.86
Lactiplantibacillus-like, log CFU/g	<2.00	6.22	6.50	6.67	<2.00	6.21	6.47	6.69

* n = 4. [1] Self-induced anaerobic fermented product. [2] Vitamin premix per kilogram: Vitamin A, 14,000,000 IU; Vitamin D3, 1,200,000 IU; Vitamin E, 44,000 IU; Vitamin K3, 2.4 g; Vitamin B1, 3 g; Vitamin B2, 7 g; Vitamin B6, 3 g; Vitamin B12, 0.04 g; pantothenate, 15 g; Niacin, 27 g; Biotin, 0.2 g; Folic acid, 2 g. [3] Mineral premix per kilogram: Cu ($CuSO_4{\cdot}5H_2O$), 15 g; Fe ($FeSO_4{\cdot}7H_2O$), 150 g; Mn ($MnSO_4{\cdot}H_2O$), 50 g; Zn (ZnO), 50 g; I (KI), 2 g; Se ($NaSeO_3$), 0.2 g.

Table S5. Analyzed composition and apparent digestibility of the basal diet in Trial 2.

**Item**	**Value**
Analyzed value	
Moisture (%)	10.2 ± 0.1
Crude protein (%)	21.3 ± 1.5
Calcium (%)	0.82 ± 0.03
Apparent digestibility	
Dry matter (%)	69.0 ± 0.4
AMEn (kcal/kg)	3339 ± 32
Crude protein (%)	65.9 ± 1.8

n = 4. Mean ± SD
